# Fibrolamellar Hepatocellular Carcinoma in the Absence of Risk Factors: A Case Report

**DOI:** 10.7759/cureus.32483

**Published:** 2022-12-13

**Authors:** Ahdab S Bawashkhah, Ghufran A Sindi, Shahad B Almatrafi, Elaf F Obaid, Reda I Bakhsh

**Affiliations:** 1 College of Medicine, Umm Al-Qura University, Makkah, SAU; 2 Department of Internal Medicine, Al-Noor Specialist Hospital, Makkah, SAU; 3 Department of Medical Oncology, Al-Noor Specialist Hospital, Makkah, SAU

**Keywords:** saudi arabia, case report, non-cirrhotic liver, chronic liver disease, hepatocellular carcinoma

## Abstract

Cancer of the liver and intrahepatic bile ducts is the sixth most frequently diagnosed cancer worldwide. In addition, primary liver cancer is the fourth leading cause of cancer-related mortality worldwide, and the second most lethal tumor after pancreatic cancer. Early diagnosis and rapid workup for the suspected case are the only paths for treating the patient with curative intent. Hepatocellular carcinoma (HCC) is usually associated with risk factors like chronic viral hepatitis and alcohol ingestion. Since HCC typically progresses silently, clinical diagnosis can be challenging, and the diagnosis may require the use of one or more imaging modalities and liver biopsy. In this case, the patient is a 29-year-old man with no risk factors, who was diagnosed early and treated without the need for a liver transplant.

## Introduction

Hepatocellular carcinoma (HCC) is the most common primary cancer of the liver. It is usually associated with chronic liver disease, caused by hepatitis B virus (HBV) and hepatitis C virus (HCV) [1]. Long-term use of alcohol is another common cause in western societies [2]. Other risk factors that increase the risk of HCC include hemochromatosis, Wilson disease, alpha-1 antitrypsin, and tyrosinemia [1-3].

HCC usually presents a long time after the growth of the tumor and comes with nonspecific liver signs and symptoms like right upper quadrant abdominal pain, jaundice, hepatomegaly, and ascites [4]. HCC presents with a non-cirrhotic liver in up to 20% of the cases, and some cases are associated with hereditary disorders or metabolic syndromes [5]. Males are more vulnerable to having non-cirrhotic HCC than females [5].

Environmental toxins may contribute to the pathogenesis of HCC; however, toxins are probably not independent risk factors but rather may act synergistically with other more common risk factors (e.g., HBV infection, aflatoxin B1, betel nut chewing, iron overload, and contaminated drinking water) [6,7]. Alcohol, tobacco, nonalcoholic fatty liver disease, diabetes mellitus, and obesity have all been associated with an increased risk of HCC as well [6-8]. There are many histologic variants of HCC including, fibrolamellar, sarcomatous, scirrhous, steatohepatitis, and clear-cell variants [9]. Fibrolamellar hepatocellular carcinoma (FL-HCC) is a rare malignant tumor that includes 1%-9% of all hepatocellular carcinomas (HCC) [10]. It is difficult to understand FL-HCC that typically affects younger persons [10]. Here we present a case of a 29-year-old man with no risk factors, who was diagnosed early with a rare type of HCC and treated without the need for a liver transplant.

## Case presentation

A 29-year-old Saudi male patient with no history of medical illness presented to the ER as a referral case from a private clinic with a one-month history of right upper quadrant pain. The pain was dull and aching in character, not radiating to any site, with no specific aggravating or relieving factors. It was associated with early satiety, complicated by nausea and vomiting for the last five days.

Physical examination revealed that the patient was conscious and oriented, had normal vital signs, and was afebrile. On abdominal examination, there was a palpable epigastric mass of 8 cm in size with a nodular surface, no ascites, and no visible veins. There was no evidence of splenomegaly or hepatomegaly, and the examination of other systems was unremarkable. Laboratory investigation results are shown in Table 1.

**Table 1:** Laboratory results

Test description	Observed value	Reference value
Complete Blood Count (CBC)		
Hemoglobin	110.00 grams per liter (g/L)	138-172 g/L
Platelet	460 x 10^9^/L	150-450 x 10^9^/L
White Blood Cell (WBC)	9.07 x 10^9^/L	4-11 x 10^9^/L
Coagulation Profile		
Prothrombin Time (PT)	16.9 sec	11-13.5 sec
Partial Thromboplastin Time (PTT)	48.1 sec	25-35 sec
International Normalized Ratio (INR)	1.32	1.1 and below
Liver Function Test (LFT)		
Aspartate Transaminase (AST)	48 Unit/Liter (U/L)	8-33 U/L
Alanine Transaminase (ALT)	21 U/L	7-55 U/L
Alkaline Phosphatase	74 U/L	44/147 U/L
Total Bilirubin	4.4 umol/L	1.71-20.5 umol/L
Direct Bilirubin	1.82 umol/L	Less than 5.1 umol/L
Lactate Dehydrogenase (LDH)	298 U/L	105-333 U/L
Albumin	26 g/L	
Amylase	56 U/L	40-140 U/L
Complete Metabolic Panel		
Blood Glucose	6.8 mmol/L	5.6-6.9 mmol/L
Potassium	3.2 mmol/L	3.6-5.2 mmol/L
Calcium	2.1 mmol/L	2.2-2.7 mmol/L

**Figure 1:**
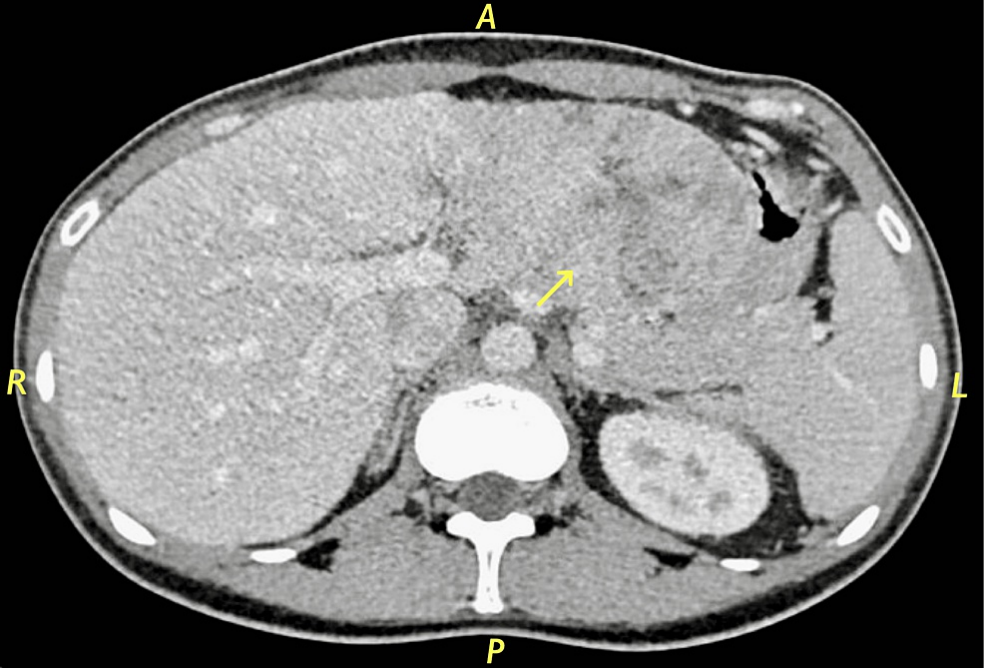
Liver triphasic CT hepatic mass consistent with fibrolamellar carcinoma

Liver triphasic computerized tomography (CT) showed a huge solitary, well-defined border and lobulated hepatic mass consistent with fibrolamellar carcinoma as shown in Figure 1.

**Figure 2:**
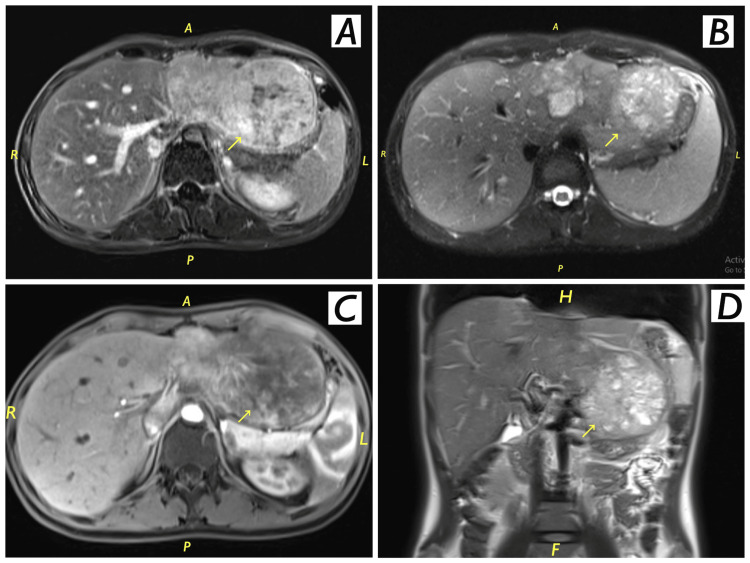
Abdominal MRI findings confirm hepatocellular carcinoma Abdominal MRI showing non-cirrhotic liver with a smooth outline; there is a large, well-defined exophytic mass with lobulated margins in the left lobe of the liver, measuring about 11.4 x 7.2 x 8.8 cm in maximum transverse, and craniocaudal view as shown in 2A, 2B, 2C and 2D.

Abdominal magnetic resonance imaging (MRI) showed a non-cirrhotic liver with a smooth outline; there was a large, well-defined exophytic mass with lobulated margins in the left lobe of the liver, measuring about 11.4 x 7.2 x 8.8 cm in maximum transverse (TR), anteroposterior (AP), and craniocaudal (CC) view, respectively, showing heterogeneous high signal intensity (SI) with low striations and central dark band in T2 WI. There were a few areas of high T1 signal intensity, likely representing hemorrhagic foci. There was heterogeneous early arterial hyperenhancement and delayed washout with faint enhancement of the central scar in the delayed phase with enhancing capsule. There were faint multifocal areas of diffusion restriction; there was no intracellular lipid; no other focal hepatic lesion, and no biliary dilation. The gallbladder was collapsed. The common bile duct (CBD) was normal. The main and right portal vein as well as the right and middle glands were unremarkable. The scanned bowel and skeleton were unremarkable as well, no free fluid or upper abdominal lymphadenopathy. The findings confirmed the suspicion of hepatocellular carcinoma of fibrolamellar type (FL-HCC) as shown in Figure 2.

Ultrasound (US)-guided biopsy of the liver mass showed a high-vascularity lesion that presented in the left lobe. Histopathological study of the liver specimen reported infiltrative neoplastic proliferation composed of sheets and trabeculae of large polygonal malignant hepatocytes with dense eosinophilic granular cytoplasm, vesicular nuclei, and prominent nucleoli, with a low index mitotic rate. Cytoplasmic pale bodies were seen, and the cord of the tumor cell was separated by lamellar fibrosis stroma (trichrome stain positive). Immunohistochemistry showed hep par 1 diffusely positive, serum alpha-fetoprotein level was negative, Ki67> 5%, and fibrinogen level was not available. CT of the chest showed no metastasis.

The decision was made to refer the patient to a hepatocellular surgeon at King Abdulaziz University Hospital. The surgeon decided to resect the whole lesion left hepatectomy with a negative margin. The resection was done after three weeks of definitive diagnosis. The surgery was successful and the mass was resected with negative margins. The patient was followed in the outpatient department (OPD) after one week, one month and three months of surgery and doing well without any complications. Follow-up CT with contrast showed no signs of recurrence.

## Discussion

The risk factors of HCC show variation according to the region which is why Asia and Africa have the highest rate of liver cancer in the world compared with the Middle East [11]. Some HCC risk factors are inheritable like alpha-1-antitrypsin deficiency and glycogen storage disease type 1. Other risk factors are usually acquired like viral infection, and chronic hepatitis B infection, which is one of the most common risk factors in Southeast Asia and Africa [12]. Hepatitis C, alcohol, obesity, and diabetes mellitus are still important risk factors [12, 13]. HCC is known to have a high association with obesity, metabolic syndrome, and iron overload [14,15]. Less than 20% of HCC cases are non-cirrhotic HCC, oral contraceptive has been associated with non-cirrhotic HCC, although rarely non-cirrhotic HCC presents with an atypical presentation [14,15].

Some studies have shown that the fibrolamellar-HCC is rare, and it affects younger age groups between 10-35 years more commonly although the older age groups are affected as well. FL-HCC is affecting both genders equally [16,17]. One of the most common characteristics of fibrolamellar carcinoma is the absence of chronic liver disease [17]. In this case, the patient is not a smoker, and he has no active liver disease, no history of hepatitis, no chronic medical illnesses, and negative family history of liver disease.

FL-HCC presents with non-specific clinical features like abdominal pain, vomiting, nausea, and early satiety [17,18]. Our patient was complaining of mild upper quadrant pain with nausea and vomiting. Generalized metastatic symptoms are uncommon in fibrolamellar carcinomas like fever, and venous thrombosis complications associated with cancer [17].

Alpha-fetoprotein (AFP) and liver transaminases are usually not elevated in FL-HCC, hence the tumor markers are not sensitive nor specific for FL-HCC, although it is used to monitor the response of the treatment [5,17]. Our patient was having normal alpha-fetoprotein, and normal alanine transaminase (ALT) but slightly high aspartate transaminase (AST).

The differential diagnosis of a large hepatic tumor from the perspective of preoperative imaging tests would include FL-HCC, HCC, hepatocellular adenoma, and focal nodular hyperplasia (FNH) [19]. Given that both HCC and FL-HCC are definite surgical indications, FNH is the most crucial differential diagnosis that must be ruled out [18]. The low signal intensity in the hepatobiliary phase of gadoxetic acid-enhanced MRI is the most important finding in differentiating FNH from FL-HCC [19]. Liver triphasic CT of the patient showed a huge solitary, well-defined border and lobulated hepatic mass consistent with fibrolamellar carcinoma. MRI of the patient showed a hypo-intense mass, non-cirrhotic liver with a smooth outline, well-demarcated, lobulated mass in the left lobe of the liver. There were faint multifocal areas of diffusion restriction, no intracellular lipid, no other focal hepatic lesion, and no biliary dilation.

Fibrolamellar carcinoma has a better prognosis than other hepatocellular carcinomas. The treatment is resection of the tumor in case of resectable mass or liver transplantation while the recurrence rate is still high reaching 60% in the first five years, so follow-up is necessary for possible re-resection in a good clinical setting [17].

## Conclusions

Fibrolamellar carcinoma is one of the histological subtypes of non-cirrhotic hepatocellular carcinoma and usually affects younger age groups and presents with non-specific hepatic symptoms and clinical features. Fibrolamellar carcinoma should be suspected in HCC diagnosed without chronic liver disease. And it could present in a healthy person without any risk factor or any liver disease with good clinical outcomes in a comprehensive clinical setting of evaluation and management.
